# Emergency department triage: an ethical analysis

**DOI:** 10.1186/1471-227X-11-16

**Published:** 2011-10-07

**Authors:** Ramesh P Aacharya, Chris Gastmans, Yvonne Denier

**Affiliations:** 1 Department of General Practice & Emergency Medicine, Tribhuvan University Teaching Hospital, Maharajgunj, P. O. Box 8844, Kathmandu, Nepal; 2 Centre for Biomedical Ethics and Law, Faculty of Medicine, Catholic University, Leuven, Belgium

## Abstract

**Background:**

Emergency departments across the globe follow a triage system in order to cope with overcrowding. The intention behind triage is to improve the emergency care and to prioritize cases in terms of clinical urgency.

**Discussion:**

In emergency department triage, medical care might lead to adverse consequences like delay in providing care, compromise in privacy and confidentiality, poor physician-patient communication, failing to provide the necessary care altogether, or even having to decide whose life to save when not everyone can be saved. These consequences challenge the ethical quality of emergency care. This article provides an ethical analysis of "routine" emergency department triage. The four principles of biomedical ethics - viz. respect for autonomy, beneficence, nonmaleficence and justice provide the starting point and help us to identify the ethical challenges of emergency department triage. However, they do not offer a *comprehensive *ethical view. To address the ethical issues of emergency department triage from a more comprehensive ethical view, the care ethics perspective offers additional insights.

**Summary:**

We integrate the results from the analysis using four principles of biomedical ethics into care ethics perspective on triage and propose an integrated clinically and ethically based framework of emergency department triage planning, as seen from a comprehensive ethics perspective that incorporates both the principles-based and care-oriented approach.

## Background

Emergency care is one of the most sensitive areas of health care. This sensitivity is commonly based on a combination of factors such as urgency and crowding [1]. Urgency of care results from a combination of physical and psychological distress, which appears in all emergency situations in which a sudden, unexpected, agonizing and at times life threatening condition leads a patient to the emergency department (ED).

The Australasian College for Emergency Medicine (ACEM) defines ED overcrowding as the situation where ED function is impeded primarily because the number of patients waiting to be seen, undergoing assessment and treatment, or waiting to leave exceeds the physical and/or staffing capacity of the ED [2]. ED overcrowding is a common scenario across the globe [1,3] and resources like staff, space and equipment are limited. Patients often have to wait for a long time before being seen by a doctor and even longer before being transferred to a hospital bed [3]. The result is not merely inconvenience but a degradation of the entire care experience - quality of care is compromised, the patient's safety may be endangered, staff morale is impaired and the cost of care increases.

The inappropriate use and/or misuse of ED services is one of the common problems leading to overcrowding [4]. Sociodemographic characteristics are predictors of nonurgent use of emergency department [5]. Public orientation [4], strengthening and expanding primary care services can be a solution to the problem [6,7].

When existing needs cannot be met by the available resources a system is needed to cope with the situation and many hospitals use a triage system in order to do this [8]. The aim of triage is to improve the quality of emergency care and prioritize cases according to the right terms [9].

The term "triage" is derived from the French word *trier *(to sort) which was originally used to describe sorting of the agricultural products. Today, "triage" is almost exclusively used in specific health care contexts [9].

Iserson and Moskop [9] describe the requirement of three conditions for triage in emergency practice:

1. At least modest scarcity of resources exists.

2. A health care worker (often called a "triage officer") assesses each patient's medical needs based on a brief examination.

3. The triage officer uses an established system or plan, usually based on an algorithm or a set of criteria to determine a specific treatment or treatment priority for each patient.

From the perspective of *ethical theories*, triage is commonly seen as a classic example of distributive justice, which addresses the question of how benefits and burdens should be distributed within a population [10]. It is traditionally used within the ethical literature as an example of a pressing ethical conflict between the utilitarian principle to do the greatest good for the greatest number, [11] the principle of equal respect for all, the principle of nonmaleficence, and the principle of non-abandonment [12].

The fundamental point of triage is the following: not everyone who needs a particular form of health care, such as medicine, therapy, surgery, transplantation, intensive care bed, can gain immediate access to it. Triage systems are designed to assist allocation decisions in this regard. These decisions are more difficult when a condition is life-threatening and the scarce resource potentially life-saving. In life threatening conditions, the question can become: "Who shall live when not everyone can live?" The crux of the matter is the seeming inappropriateness of abstract allocation principles at the level of face-to-face relationships. The general utilitarian concerns of the system, which in the context of scarcity comes down to calculating and choosing between patients on the basis of abstract reasoning (focused on "statistical lives", realizing the best results out of an abstract cost-benefit analysis applied to patients as abstract cases), seems to collide with the Hippocratic duty of doing as much as you can for the patients who need care (focused on "identifiable lives", that is, on the patients as particular persons with whom one stands in a face-to-face care relationship) [12].

Ethical issues are hardly considered in emergency department setting. A study by Anderson-Shaw et al has suggested that patients hospitalized through ED often present with ethical dilemmas significantly impacting their inpatient care and overall health outcomes [13]. There is need of more research regarding the proactive use of ethics consultation in ED.

Within existing *medical literature*, the controversies relating to the ethics of triage in medical practices predominantly date back to the early eighties [14]. Recent studies focus on the contemporary concept of triage [9], underlying values and preferences [10], evolution of systems [15] and their variation according to traditions, cultures, social context and religious beliefs [16], update on guidelines [17] and position statements [18].

Currently, the existing literature on triage is deficient in two ways. Either there is a predominant focus, from a medical perspective, on the practical elements of triage and on clinical-based guidelines. Or there is a focus, from an ethical perspective, on the domain of distributive justice, with its conflicting principles, as such remaining on the abstract level of reasoning. The aim of this paper is to bring the two strands together.

The central question is the following: how can triage systems in emergency care be ethically assessed, so as to realize optimal use of scarce resources in an ethically just way without remaining on the abstract level, that is by taking the effect of triage on the individual patients and caregivers into account?

In order to do this, we will focus on ED triage. We aim at complementing existing literature on ED triage with an ethical framework that can help ED management teams in planning and executing triage for the care of emergency patients in the daily practice.

### Triage in Health Care

Common contexts of triage in contemporary health care practices are pre-hospital care [19], emergency care, intensive care (who to admit), waiting lists (e.g. for lifesaving treatments such as organ transplants) and battlefield situations [20]. In case of emergencies and disasters, three stages of triage have emerged in modern healthcare systems [15].

1. First, pre-hospital triage in order to dispatch ambulance and pre-hospital care resources.

2. Second, triage at the scene by the first clinician attending the patient.

3. Third, triage on arrival at the hospital ED.

During the last decade, the issue of pandemic triage has entered the discussion of triage [21-23]. The emerging infectious disease like Severe Acute Respiratory Syndrome (SARS) and Pandemic Influenza have alerted emergency departments to the need for contingency plans. This applies to triage for intensive care services as well. In such public health emergencies, the managerial emphasis shifts from the individual to the population, from "individual" to "statistical" lives, trying to realize a maximal outcome out of the available resources [24]. Nevertheless, emergency staff continues to be confronted, on a face-to-face level, with the care for individual patients in need, whom they might not be able to help.

### Emergency Department Triage

Triage is a system of clinical risk management employed in emergency departments worldwide to manage patient flow safely when clinical needs exceed capacity. It promulgates a system that delivers a teachable, auditable method of assigning clinical priority in emergency settings [17].

In contemporary emergency care, triage is regarded as an essential function not only during massive influx of patients as in disasters, epidemics and pandemics but also in regular emergency care departments. The burden in emergency care is increasing and so are the expectations of patients [1]. In hospitals that apply triage for regular emergency care, triage is the first point of contact with the ED. Assessment by the triage officers involves a combination of the chief complaint of the patient, general appearance and at times, recording of vital signs [25].

### Guidelines for Emergency Department Triage

Triage guidelines score emergency patients into several categories and relate it to the maximum waiting time based on specific criteria of clinical urgency. Initial versions of triage guidelines had three levels of categorization mostly termed as emergent, urgent and non-urgent [25]. Studies have revealed that five-level triage systems are more effective, valid and reliable [25,26]. In contemporary emergency care, most triage systems sort out patients into five categories or levels (Table 1) including the time within which the patient should be seen by the emergency care provider [27].

**Table 1:** Five-level Triage Systems

System	Countries	Levels	Patient should be seen by provider within
**Australasian Triage Scale (ATS)**	Australia	1 - Resuscitation	Level 1 - 0 minutes
	New Zealand	2 - Emergency	Level 2 - 10 minutes
		3 - Urgent	Level 3 - 30 minutes
		4 - Semi-urgent	Level 4 - 60 minutes
		5 - Nonurgent	Level 5 - 120 minutes

**Manchester**	England	1 - Immediate (red)	Level 1 - 0 minutes
	Scotland	2 - Very urgent (orange)	Level 2 - 10 minutes
		3 - Urgent (yellow)	Level 3 - 60 minutes
		4 - Standard (green)	Level 4 - 120 minutes
		5 - Nonurgent (blue)	Level 5 - 240 minutes

**Canadian Triage and Acuity Scale(CTAS)**	Canada	1 - Resuscitation	Level 1 - 0 minutes
		2 - Emergent	Level 2 - 15 minutes
		3 - Urgent	Level 3 - 30 minutes
		4 - Less urgent	Level 4 - 60 minutes
		5 - Nonurgent	Level 5 - 120 minutes

Table 2-2 Five-level Triage Systems

**Figure 1:**
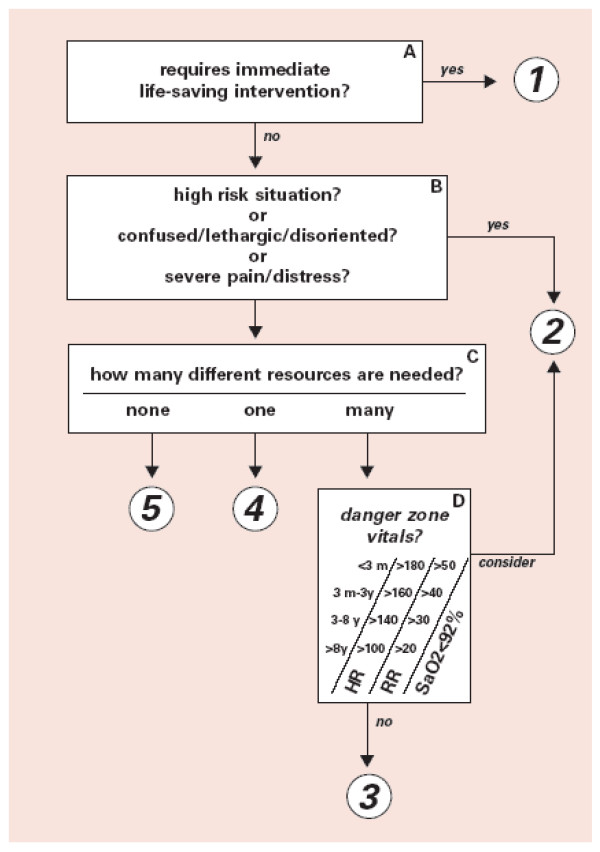
**Emergency Severity Index (ESI) Triage Algorithm, v. 4 (Five Levels)**.

The most commonly used guidelines for ED triage on the international literature are *The Manchester Triage Score *[17,28,29], *The Canadian Triage and Acuity Scale *[28-31], *The Australasian Triage Scale *[28,32] and *Emergency severity Index *[27,29]. In ESI, there are five-levels of these triage score (see Figure 1). In addition national and institutional guidelines are also developed and used in practice [15,33].

When reflecting on the question whether these triage systems say anything about *how *to sort a patient among one of the five levels, we can apply *The Manchester Triage Score *[17] as an example. This triage system selects patients with the highest priority first and works without making any assumptions about diagnosis. In this method the actual priority is determined by using flow charts which utilizes 'discriminators' at each level of priority. Discriminators are factors (general or specific) that discriminate between patients to be allocated to one of the five clinical priorities. There are six *general *discriminators for triage: life threat, haemorrhage, pain, conscious level, temperature and acuteness. These have to be practiced at each level of priority and it is essential for the triage officer to understand the triage method. For example: Pain can be *severe pain, moderate pain and recent pain. Specific *discriminators are applicable to individual presentations or to small groups of presentations, which tend to relate to key features of particular conditions. For example: *cardiac pain *or *pleuritic pain*. Thus, the specific criteria of triage are based on clinical urgency.

Though terminology of categorization differs slightly between the various guidelines, their practical meaning is more or less the same. Triage is a brief encounter between triage officer and patient, which takes two to four minutes [34]. Subsequently, the patient is labeled with a colored tag. Depending on this tag, the patients are sent to specified areas where they will be consulted by the physicians. While undergoing treatment, the patient may improve or worsen and so may need to be re-triaged and shifted to appropriate area for further treatment. Thus, triage is a continuous process in which clinical characteristics need to be checked regularly to ensure that the priority remains correct.

**Figure 2:**
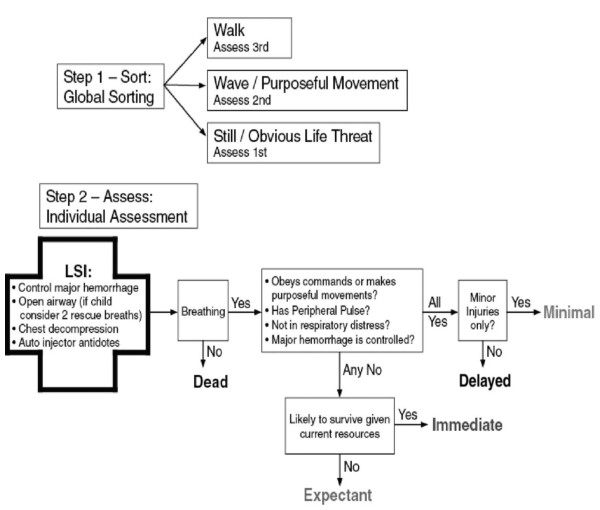
**SALT triage scheme**. LSI = Life Saving Interventions.

**Figure 3:**
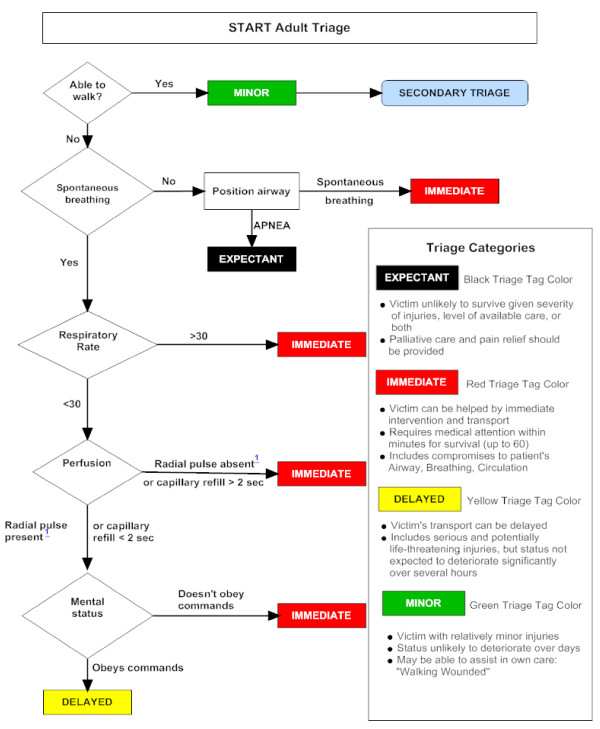
**START Triage algorithm for adult patient**. Adapted from http://www.start-triage.com/

The Canadian Triage and Acuity Scale (CTAS) consist of separate guidelines for adult [30] and child [31] patients. In The Manchester Triage Score [17], the level of consciousness in adult and children is considered separately. A guideline, entitled SALT (sort, assess, life-saving interventions, treatment and/or transport) triage, was developed in 2008; which incorporates aspects from all of the existing triage systems (see Figure 2) to create a single overarching guide for unifying the mass casualty triage process across the United States [35]. START triage utilises the use of colours green, yellow, red and black to categorise the patients (see Figure 3). More importantly, separate guidelines have been developed for potential pandemics like influenza [22,23] and special situations like the use of weapons of mass destruction and bioterrorism [36]. During sudden emergence of '2009 H1N1 influenza', web-based self-triage named Strategy for Off-Site Rapid Triage (SORT) was disseminated by H1N1 Response Centre to reduce a potential surge of health system utilization without denying needed care [37].

The Sacco Triage Method (initially known as resource-constrained triage method) is an evidence based outcome driven triage which considers the resources to maximize the expected survivors. Triage decisions are based on a simple age adjusted physiological score (i.e. respiratory rate, pulse rate and best motor response) that is computed routinely on every trauma patient and are correlated to survival probability [38].

## Discussion

ED triage introduces several ethical questions, which have received less attention in the general literature on triage. Below, we will carry out an ethical analysis by firstly applying the four principles of biomedical ethics developed by Beauchamp and Childress [9]. Then, we will look at the ethical aspects of ED triage from the care ethics perspective, an influential ethical theory [39-42] that evolved out of the works of Carol Gilligan [43] and Joan Tronto [44].

### The Principle-based Approach

#### Respect for Autonomy

Respect for autonomy is a pivotal criterion for decision-making in health care and provides that competent persons have the right to make choices regarding their own health care. Respect for patient autonomy became especially important with the emancipation of the patient in the socio-political context of democracy and the human rights movement. It resulted in the decline of the paternalistic relationship between a doctor and patient and encouraged individuals to protect their personal values. To respect an autonomous agent is, at a minimum, to acknowledge the person's right to hold views, to make choices, and to take actions based on personal values and beliefs. As Beauchamp and Childress state, such respect involves *action*, not merely a respectful *attitude *[12]. It involves actively treating persons to enable them to act autonomously.

While considering ED triage, autonomy is very difficult to assess especially when urgent situations arise. Here, it is important to find out who decides about the emergency of a situation in the first place.

Let us first look at the viewpoint of the patient. The American College of Emergency Physicians defines emergency services as follows: [45]

"Emergency services are those health care services provided to evaluate and treat medical conditions of recent onset and severity that would lead a prudent lay person, possessing an average knowledge of medicine and health, to believe that urgent and/or unscheduled medical care is required."

According to this definition, urgency is determined by a lay person and emergency services have two components; firstly evaluation and then, treatment. Most of the patients who come to an emergency department believe they have a problem requiring immediate medical care. In such cases, ED triage raises ethical questions particularly when the emergency service is being denied. One can consider triage as an evaluation, although technically it is not a complete medical evaluation. Refusal to provide emergency treatment to a patient presenting to the ED contradicts to the principle of respect for autonomy. The triage officer takes the decision without consent of the patient which can be regarded as the paternalistic approach of decision making. A study [46] published in 1994 on refusal of emergency care showed that among 106 refused patients, 35 (33%) had appropriate visits and four of them had to be hospitalized. Refusal was based on the triage guidelines which mentioned 'non-emergency complaints' so the author concluded that the guidelines were not sufficiently sensitive. Thus, such refusal to emergency treatment conflicts not only with the principle of respect of autonomy but also with the demands of good quality care in emergency services.

When looking at the viewpoint of the care provider, we see that the decisions are being made by the triage officer or the concerned authority of the ED. Triage is the initial step in the evaluation of a patient's complaint(s) before initiating medical evaluation and management and generally, informed consent is not considered as a part of triage process [17]. In addition, there is exemption from informed consent requirements even for emergency research [47]. Emergency treatments can be given under the doctrine of necessity if an adult patient lacks capacity to give consent [48]. Given the urgent character of emergency situations, respect for autonomy in the form of informed consent is often not the first ethical priority, which is perfectly normal because the urgency of the situation does not provide room for it. In such situations, the necessary care should be provided instantly.

Nevertheless, the fact that informed consent cannot factually be realized in many ED situations does not mean that respect for autonomy cannot be taken into account at all here. Davis et al reported that even acutely ill emergency patients preferred respect for autonomy in medical decision making and increasing acuity of illness at presentation does not predict a decreased desire for autonomy [49].

An important way of respecting autonomy as much as possible here is by focusing on good and clear ED communication. To exercise respect for autonomy, health care workers must be able to communicate well with their patients. However, the emergency department (ED) presents unique challenges to effective provider-patient communication, such as lack of privacy, noise, frequent interruptions, and lack of an established medical relationship. A study on ED communication concluded that the physician-patient encounter was brief and lacking in important health information such as specifying symptoms that should prompt return to the ED [50]. Good communication requires, most importantly, listening as well as talking and is usually necessary for giving patients information about the proposed intervention and for finding out whether patients want that intervention [51]. Triage officers should routinely inform patients about their triage level, and their estimated waiting time before being seen by the doctor [52].

However, the common triage guidelines have not considered communication skills and informed consent as part of triage procedure [17,27]. Effective communication is not a function of time but rather one of skill. Few extra seconds spent on each tasks are actually time efficient and can decrease inappropriate workup, interpersonal conflict, and litigation, and can enhance compliance with follow-up care [53]. Thus, though the time factor is generally blamed for this, this should not necessarily be the case because good communication can be part of the triage process itself. As such, respect for autonomy may be realized as much as possible in ED situations.

#### Nonmaleficence

The principle of nonmaleficence can be described as "do no harm". The Hippocratic Oath mentions this obligation as "I will use treatment to help the sick according to my ability and judgment, but I will never use it to injure or wrong them" [12]. One ought not to inflict evil or harm. Harm is not directly inflicted by triage except when hopelessly injured patients are considered in the dead category. Even during disasters, under given circumstances; health care professionals are always obligated to provide the reasonably best care. The aim is to secure fair and equitable resources and protections for vulnerable groups [54].

Waiting long for a consultation can increase pain and suffering and, at times, worsen the outcome and thus, result in indirect harm. Psychosocial harm includes stress, fear, feeling neglected or not being taken care of.

Triage guidelines aim to avoid harm to the patient by sorting the patients as quickly and efficiently as possible. However, in emergency care, especially in situations of overcrowding, treating one patient might threaten the welfare of another patient by not being able to take care of both. Studies in different centres have found an association between overcrowding and reduced access to care, decreased quality measures, and poor outcomes [55].

Sometimes, referral to other centres can result in more quick and effective service and thus, harm in the form of excessive delays may be avoided [18]. Furthermore, medical care is not only the diagnosis and treatment in emergency care; patients value effective communication and short waiting times over many other aspects of care [56]. Lack of communication of triage times and categories is one of the causes of aggression and violence of patients and accompanying persons towards emergency staff [57]. Crilly et al. reported around 67% of patients who exhibited violent behaviour either did not wait for treatment or had been in the emergency room for less than one hour [58].

Ekwall et al. suggest the importance of addressing the psychosocial needs of patients of varying levels of urgency through their social interactions at triage [59]. Existing triage guidelines [17,27] miss to incorporate this aspect of care, which can compromise the principle of nonmaleficence.

#### Beneficence

Beneficence is a moral obligation of contributing to the benefit or well-being of people and thus is a positive action done for the benefit of others instead of not merely refraining from harmful acts. The norms of the principle of beneficence are as follows [12]:

1. One ought to prevent evil or harm.

2. One ought to remove evil or harm.

3. One ought to do or promote good.

Health care providers in the ED have an ethical obligation to attempt to provide benefits to the patients by taking their complaints seriously and by managing their problems according to prevailing standards of care. By applying a system of triage, they seek to improve the quality of care by using the available resources as effectively and efficiently as possible. The ultimate goal of triage is to preserve and protect endangered human lives as much as possible by assigning priority to patients with an immediate need for life-sustaining treatment. Though due consideration should be given to the available resources, the life and health of patients is priority.

In triage, tendency of overtriage particularly in patients with trauma may be a tendency for beneficence. However, it is an "err on the side of caution". Overtriage not only increases the cost of medical care [60] but also may result in worse outcome [61,62].

Nevertheless, this has to be done in a context characterized by urgency, overcrowding, and limited medical resources (time, staff, medical equipment, drugs etc), which increases the pressure upon health professionals in the ED. In the same line of reasoning, triage officers mention the fear that an incorrect triage category allocation may lead to a delay in treatment and at worst, the death of a patient, particularly when waiting times are long [63].

#### Justice

Justice, more specifically understood as distributive justice, requires that given limited resources, allocation decisions must be made *fairly*, and that benefits and burdens are distributed in a just and fair way [12]. Triage schemes systematically allocate the benefits of receiving health care, and the burdens of limited, delayed, or deferred care, among a population of sick or injured persons [10]. This does not mean that each person or group must get an equal share of the scarce resources (equality), but rather a fair share based on *appropriate *criteria and principles (equity) [18].

Generally, the criteria and principles relevant for triage in emergency care can be classified into three general categories, among which a balance has to be created [1,64]. The first principle is the *principle of equality*. It is based on the idea that each person's life is of equal worth and holds that everyone should have an equal chance to receive the necessary care. A triage system based on this principle would presumably operate on a first-come, first served basis [16], giving equal consideration to all, no matter how resource intensive one's treatment will be, or even though the care for one or a few patients may result in a greater burden for many [10]. The reluctance of physicians to abandon any patient whom they believe they can save may give implicit support to this type of triage. It is also known as the rescue-principle or the principle of non-abandonment [65]. However, giving priority to the principle of equality in emergency [10] care situation is not an optimal strategy to realize efficient use of scarce resources.

The *principle of utility*, on the other hand, holds that actions should be judged by their consequences and how far they produce the greatest net benefit among all those affected. Or put simply, to do the greatest good for the greatest number. In fact, utilitarianism is the rationale for triage systems, insofar as they seek to use the available but scarce medical resources as efficiently as possible [11]. In itself, however, the principle of utility remains silent with regard to which goods or benefits are to be maximized [23]. In order to produce the greatest net benefit, we must have a clear account of which kinds of benefit are to be promoted. For instance, triage systems may seek to achieve the health benefits of survival (saving the most lives), restoration or preservation of function (by maximizing quality-adjusted life-years or disability-adjusted life-years), relief of suffering, and so on [10,23]. To maximize the chosen benefits overall, however, triage systems may dictate that treatments for some patients be delayed, often resulting in poorer outcomes for those patients. Bad consequences for some may be justified if an action produces the greatest overall benefit. Triage systems recognize this because in emergency situations, the resources are scarce in relation to the needs of the patients. Consequently, the needs of some patients will be subordinated to those of others in order to maximize utility. Which one of the criteria will, in fact, maximize utility, depends on complex empirical aspects of the situation and on the triage officer's assessment capacities.

One particular criterion, however, is being reflected in the third principle of justice, i.e. *the principle of priority to the worst-off*. Here, much depends on how one defines the worst-off group. Are they the most needy? The most urgent cases? Or the ones with the lowest prospects? Or even the poor and disenfranchised people who most often use the emergency departments because they have no other choice of receiving health care? [18] Suppose the worst possible outcome would be death [66]. Accordingly, the worst-off group would be the severely ill or injured people whose risk of death is highest, and for whom the likelihood of successful treatment is low, i.e. the ones at the edge of life and death. Guided by this principle, triage systems would give priority to treatment of this clearly disadvantaged group. However, it would be highly inefficient if maximizing the benefits to this group would imply investing a disproportionate share of scarce resources into a group of patients who are not likely to survive. Consequently, a correction has to be made. Proponents of this principle would probably focus on minimizing the number of avoidable deaths by directing the triage system to focus on the "salvageable" patients [10].

#### What do we learn from this?

Let us take stock. How can good-quality care be given in urgent situations, with limited resources, in an overcrowded ED? By applying a triage system, one can quickly and efficiently sort patients according to clinical priority, thus aiming to manage patient flow safely when clinical needs exceed capacity. The triage process happens during the period between the time patients first present in the ED and the time at which they are first seen by a doctor [3]. Even though it is a quick and seemingly impersonal system of sorting patients, it has great impact on people and on the quality of emergency care. On the basis of the above-made principle-based analysis, we have reached some general insights into the ethical aspects of that impact. From the four principles of biomedical ethics (autonomy, nonmaleficence, beneficence, and justice), we can derive the following areas of special attention:

(1) The principle of respect for autonomy, especially in ED situations, is very difficult to assess, most particularly when urgent situations arise, as often is the case. Special attention is needed for particular ways of respecting autonomy as much as possible, for instance by appropriate and adequate communication during the triage process.

(2) The principle of nonmaleficence is under pressure since triage can reinforce the physical (long waiting times, increasing pain and suffering, deteriorating condition) and psychological harms (stress, fear, feeling neglected) that come with the underlying pathological conditions.

(3) Aggression and violence are common phenomena in the ED. They aggravate the working conditions, impair staff morale and complicate people's abilities to make proper decisions. The principle of beneficence is compromised by the pressure upon health professionals, which in turn reinforces their feelings of fear for making wrong decisions [63].

(4) With regard to the principle of justice, it is finally a continuous assignment to check whether the system realizes a fair balance between the principle of equal respect for all and efficient use of resources. Here, it is important to see whether the just situation can be realized in a human way.

The results from this ethical analysis, based on the four principles of biomedical ethics, are interesting but insufficient since they do not offer a *comprehensive *ethical view for two reasons: (1) they only offer fragmented pieces of the triage puzzle; and (2) they do not provide a view on the dynamics of the care process. To address the ethical issues of ED triage as seen from a more comprehensive ethical view, the care ethics perspective might offer additional insights.

### The Care Ethics Perspective

Care ethics is an ethical theory that evolved out of the Kohlberg-Gilligan debate on moral psychology and from the work done by social scientists, such as Joan Tronto in the USA and Selma Sevenhuijsen in the Netherlands [43,44,67]. According to this theory, care has important ethical value, not only within our own particular daily lives, but also within the societal context of education and social policy. As for health care ethics, the care perspective has until now been primarily applied in the fields of nursing [68,69], care for elderly people [70], mental health care [71], prenatal diagnosis and abortion [72,73], care for people with disabilities [74,75] and care for people suffering from dementia [76]. As such, the care ethics perspective has become a very influential viewpoint within ethical theory [39].

In this paper, we will apply the care ethics perspective to the issue of ED triage because we are convinced that the care ethics perspective offers important ethical insights into the dynamic character of triage within the setting of emergency care. By focusing on the dynamic aspects of delivering acute medical care, it provides an important addition to the predominantly fragmented principle-based approach. Here, we opt for an ethical analysis according to the four dimensions of care, as developed by Joan Tronto [44].

#### Four Dimensions of Care

In her pioneering book *Moral Boundaries *(1993), Joan Tronto distinguishes four dimensions of care, each comprising a corresponding ethical attitude [44,77]. The four dimensions of care can help us to understand the ethical meaning of ED triage as a fundamental part of the entire care process.

The first dimension, *'caring about'*, is the starting point of care and refers to being concerned about the condition of a person and paying attention to the vulnerability of this person confronted with. The corresponding ethical attitude is *attentiveness *and refers to the actual recognition of a need that should be cared about.

In triage, the ethical attitude of attentiveness to the needs of people, respecting their autonomy, even within the brief examination by the triage officer, is the starting point of the process and is important for ensuring that people are not being neglected. This is also a continuous attitude, for a patient may need re-triaging due to worsening or improvement of condition, or may suffer from psychological distress, due to long waiting times and lack of information.

The second dimension is *'taking care of'*. It refers to assuming the responsibility for providing the necessary care. The challenge to improve the patient's condition is recognised. Here, *responsibility *is the corresponding ethical attitude.

The triage officer takes up the responsibility to improve the patient's condition as much as possible. This means that he tries to make the right decisions in order to guarantee that the patient will be cared for as well as possible, given the circumstances of scarcity of resources.

*'Actual care giving' *is the third dimension of care and refers to the effective and adequate way to meet the patient's needs. This dimension of care requires the necessary *competence *to provide the actual care in a professional way.

By sorting patients competently, triage functions as a necessary part of good-quality emergency care. From a care ethics perspective, competent triage not only comprises the medical competence of sorting patients according to criteria of clinical urgency, but also includes attention to proper communication and respect for the patient's privacy, thus avoiding psychological harm.

Good care requires feedback and verification that the patient's needs are actually being met. This brings us to the final dimension of care, namely that of *'care receiving' *and the corresponding attitude of *responsiveness*, which refers to the response of the patient to the given care.

The dimension of care receiving is mostly lacking in the practice of triage and at times leads to conflict. Nevertheless, checking to see how the given care is being received is very important since the decisions made by the triage officer can have potential negative impact on patient's condition (e.g. patient's safety may be endangered or their condition may deteriorate) and on their experiences (distress, fear, anger). The result is not merely inconvenience but rather a degradation of the entire care process. As such, and in combination with the attitude of attentiveness, the triage officer needs to seek the responsiveness of the patient, which helps to address ethically relevant issues like respect for autonomy and the issue of informed consent, lack of communication, lack of privacy and psychological harm.

#### Framework of Interpersonal Relationships

Care practices always take place within a framework of interpersonal relationships, where the caregiver(s) and the care receiver are reciprocally involved in a dynamic interaction of giving and receiving care [41]. Reciprocity consists of verifying that the given care meets the patient's needs, thus avoiding the risk of paternalistic or inadequate care.

In his theoretical study, Gastmans points at the fact that the characteristics of relatedness and reciprocity should also be understood against the background of a very particular social context [41,77]. Applied to ED triage, we can point at the way in which the reception of people is being organized and at the way in which people in need are being approached in their first contact with the ED staff. The way in which people are being received and taken care of when entering the ED, their contact with the triage officer, are important parts of the particular care process, because they are the first encounters between patients, their relatives, caregivers and the hospital, and often the starting point of an overall care process.

#### Institutional Framework

In general, care ethics is mainly considered as an ethics of individual relationships [39]. However, care practices should always be considered against a broader horizon of social practices as a whole. The crux of the matter is that the care ethics perspective looks at care in ethical terms; at the ethical meaning of care. If we want to do this properly, we always also have to look at the specific institutional context within which care is actually being provided. This context (for instance the specific hospital culture, and its ways of dealing (or not dealing) with ethical issues regarding care) can be obstructive or supportive to the kind of care that can be given. Without sufficient attention for these contextual determinants of care, the care ethics perspective can only provide ethical analyses of care that seem very guilt-inducing for the particular care providers.

Accordingly, a careful interpretation of ED triage makes clear that a relationship between care professionals and patients cannot be seen as isolated interactions. They are always situated in a broader care process, which is enacted in the teamwork of caregivers, being part of a particular health care institution, which may have (or may not have) a carefully developed policy on ED triage [41].

Moreover, the process and outcome of ethically sensitive decision-making processes in ED triage is influenced, not only by institutional factors, such as the presence of policies, but also by the ethical culture of the hospital as organization [78], as it manifests itself in the working relationships within the team and within the hospital, in the professional atmosphere, in hierarchical relationships, etc. For instance, ethically sensitive decision-making in ED triage implies that hospital management provides sufficient support for the ED staff, both with regard to training, for instance on communication skills and aggression management as well as with regard to feedback and psychological support.

Ethical problems in hospitals often occur in an atmosphere of powerlessness, (in)efficiency, problems of cost-effectiveness, pressure, (in)competence, scarcity of human and financial resources, etc. It is this institutional and professional atmosphere, which determines what ethical problems are being expressed and how they are being dealt with in the hospital. Hence the importance of developing ED triage *as part of *a hospital-wide strategy for fixing ED overcrowding [3]. Such a hospital-wide strategy requires cross-departmental and cross-role coordination at all times.

## Summary

In this paper, we have identified the ethical dimensions of ED triage, which provide the moral framework for decisions made by triage officers. In order to carry out their task effectively, it is essential that hospitals engage in emergency department triage planning. Different from triage systems, that are exclusively clinical-based and narrowly focused on the ED, it is important to opt for an integrated clinically and ethically based form of triage planning, as seen from a comprehensive ethics perspective that incorporates both the above-described principles and care-oriented approach. Such a way of ED triage planning would incorporate the following characteristics.

(1) From the complementary dialogue between the principle-based approach and the care-oriented approach, we can conclude that a clinically and ethically based ED triage process is not only based on a momentary decision made by one person. It also takes relevant ethical principles as respect for autonomy, nonmaleficence, beneficence, and justice into account, as well as the fact that triage is a part of dynamic care process incorporating the four dimensions of care.

(2) Based on the essential importance of a supportive institutional framework, it is essential to opt for a hospital-wide strategy of triage planning with a broad involvement of relevant people. Hospital management, ED management and staff, triage officers, directors and staff of other departments are important stakeholders in the process [3,10]. As triage involves significant moral implications, it is important to involve public representatives and ethics scholars in the development of institutional ethics policies on triage planning [10].

(3) Just as triage itself is a dynamic process, and in itself part of the dynamic process of overall patient care, it is important to consider triage planning as a phenomenon that is susceptible to change. Hence, it is important to carry out regular reviews of the hospital's ED triage protocol, based on experiences of staff and patients, and on evolutions in care [10]. Proposed revisions of the protocol could then be reviewed and evaluated by multidisciplinary task forces, hospital ethics committees, or by organizations of emergency medicine and nursing professionals, according to its compliance with the comprehensive ethics perspective that incorporates both the above-described principles and care-oriented approach.

(4) ED staff has to operate in highly stressful, ethically sensitive, and sometimes even traumatic circumstances. Providing sufficient support on educational (communication, stress and aggression management), psychological (feedback) and ethical level, is essential for realizing a clinical-ethical based process of triage planning. A good and supportive hospital culture is a crucial determinant for this.

As such, the various ethical aspects that are intrinsically related to ED triage, and which we have identified by our ethical analysis, can help to create a supportive clinical-ethical framework for ED triage.

## Abbreviations

ACEM: Australasian College for Emergency Medicine; ATS: Australasian Triage Scale; CTAS: Canadian Triage and Acuity Scale; ED: Emergency Department; ESI: Emergency Severity Index; LSI: Life Saving Interventions; SALT: Sort, Assess, Life-saving interventions, Treatment and/or transport; SARS: Severe Acute Respiratory Syndrome; SORT: Strategy for Off-Site Rapid Triage; START: Simple Triage and Rapid Treatment;

## Competing interests

The authors declare that they have no competing interests.

## Authors' contributions

RPA conceptualised the topic, designed ethical analysis, collected documents and wrote major parts of the manuscript. CG linked the ethical analysis of emergency triage with care ethics and also revised the manuscript. YD guided to design the ethical analysis, contributed on the distributive justice and revised the manuscript at several stages of preparation. All authors read and approved the final manuscript.

## Pre-publication history

The pre-publication history for this paper can be accessed here:

http://www.biomedcentral.com/1471-227X/11/16/prepub
